# Perspective and Prospects for Ordered Functional Materials

**DOI:** 10.1002/advs.202300193

**Published:** 2023-03-08

**Authors:** Hai‐Tian Zhang, Tao Zhang, Xiangyi Zhang

**Affiliations:** ^1^ School of Materials Science and Engineering Beihang University Beijing 100191 China; ^2^ State Key Laboratory of Metastable Materials Science and Technology Yanshan University Qinhuangdao 066004 China

**Keywords:** Materials design strategy, ordered microstructures, magnetic materials, catalytic materials, thermoelectric materials

## Abstract

Many functional materials are approaching their performance limits due to inherent trade‐offs between essential physical properties. Such trade‐offs can be overcome by engineering a material that has an ordered arrangement of structural units, including constituent components/phases, grains, and domains. By rationally manipulating the ordering with abundant structural units at multiple length scales, the structural ordering opens up unprecedented opportunities to create transformative functional materials, as amplified properties or disruptive functionalities can be realized. In this perspective article, a brief overview of recent advances in the emerging ordered functional materials across catalytic, thermoelectric, and magnetic materials regarding the fabrication, structure, and property is presented. Then the possibility of applying this structural ordering strategy to highly efficient neuromorphic computing devices and durable battery materials is discussed. Finally, remaining scientific challenges are highlighted, and the prospects for ordered functional materials are made. This perspective aims to draw the attention of the scientific community to the emerging ordered functional materials and trigger intense studies on this topic.

## Graphical abstract

Structural ordering is an emerging nature‐inspired materials design principle to achieve mutually exclusive properties in functional materials. By rationally constructing ordered structural units and their characteristics, many new phenomena and superior properties beyond those of existing materials are discovered in the emerging ordered functional materials that are critical to high‐efficiency energy technologies.

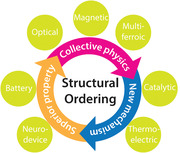

## 1 Introduction

Advanced functional materials with enhanced properties are essential to transformative technologies,^[^ ^1^, ^2^, ^3^ ^]^ such as electric cars, smart devices, and emerging artificial intelligences. However, after decades of study, the performances of many functional materials are approaching their limits as conflicting properties are often simultaneously required.^[^ ^4^, ^5^, ^6^ ^]^ For example, permanent magnetic materials that are critical to renewable energy technologies have reached their energy density ceiling,^[^ ^1^ ^]^ owing to a trade‐off between saturation magnetization and coercivity that are both needed for high energy density.^[^ ^7^ ^]^ On the other hand, there are high demands for better properties in functional materials across a range of applications, from flexible displays and wearable sensors to highly efficient energy generation and saving technologies.^[^ ^2^, ^3^, ^8^ ^]^ The unmet needs highlight the scientific challenges of how to create functional materials with amplified properties and new functionalities via transformative strategies.

In general, materials are classified into natural materials and artificial materials, i.e., man‐made materials,^[^ ^9^, ^10^ ^]^ but they adopted completely different development routes. Artificial materials emerged in the prehistoric Bronze Age and Iron Aged and developed quickly through creating or discovering new compounds by compositional design.^[^ ^9^, ^11^ ^]^ On the contrary, natural materials took a way of structural design, through building complex, multiscale, and hierarchically organized structures with a fairly limited number of components over the last billions of years.^[^ ^9^, ^11^ ^]^ Such organized structures, e.g., ordered structures, lead to unusual and often remarkable properties of natural materials. For example, both bamboo and wood are light weighted and exhibit unique combinations of high strength and high toughness that are inaccessible in artificial materials available today.^[^ ^6^ ^]^ Moreover, the feathers of a peacock have delicate and ordered structures across multiple length scales, making them both robust and light weighted.^[^ ^10^ ^]^ As such, bioinspired strategy provides us a transformative route to design structure‐ordered materials with emergent functionalities and enhanced properties not found in traditional man‐made materials.

Indeed, by rationally engineering ordered structures with structural units, e.g., constituent atoms, components/phases, grains, twins, and domain structures, as well as their characteristics such as size, concentration/content, and orientation, new phenomena and superior properties were discovered in the ordered materials across mechanical, catalytic, thermoelectric, and magnetic materials.^[^ ^6^, ^12^, ^13^, ^14^, ^15^, ^16^, ^17^, ^18^ ^]^ These achievements initiate an emerging field of ordered materials, in contrast to the conventional materials with a random mixture of constituents. The ordered structures can be created at different length scales, yielding atomic‐, nano‐, micro‐ and macro‐scale structural orderings as well as multiscale structural orderings across multiple length scales.^[^ ^19^ ^]^ The vast structural diversity of ordering with the abundant structural units, far beyond the reach of natural materials,^[^ ^9^, ^20^ ^]^ opens up unprecedented opportunities to create transformative materials, as the unique ordering among these structural units may activate collective behaviors to realize amplified properties and/or additional functionalities unattainable in existing man‐made materials,^[^ ^3^, ^21^ ^]^ in which the structural units are often distributed randomly and thus leads to a homogeneous structure.

**Figure 1:**
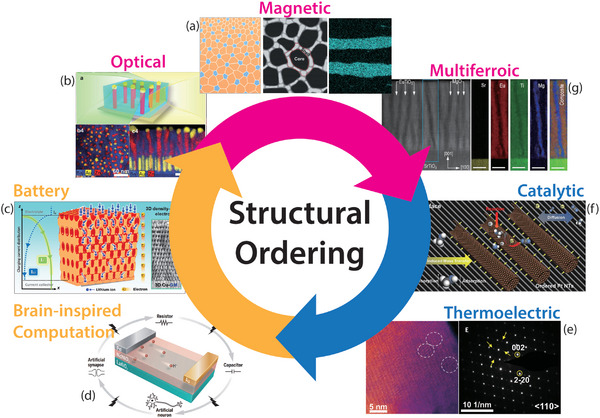
Structural ordering in functional materials. While still in the nascent stage, the strategy of structural ordering has been successfully applied to several important research fields in functional materials such as optical, magnetic, multiferroic, catalytic, and thermoelectric materials. In addition, recent studies discover that ordered structures can lead to transformative functionalities in battery materials and brain‐inspired computation. Through introducing exotic physical mechanisms not accessible for conventional materials with random and disordered microstructure, structural ordering provides a new gateway to design future functional materials. a) Reproduced with permission.^[^ ^17^ ^]^ Copyright 2021, John Wiley and Sons. Reproduced with permission.^[^ ^47^ ^]^ Copyright 2016, American Chemical Society. Reproduced with permission.^[^ ^45^ ^]^ Copyright 2018, John Wiley and Sons. b) Reproduced with permission.^[^ ^31^ ^]^ Copyright 2018, John Wiley and Sons. c) Reproduced with permission.^[^ ^59^ ^]^ Copyright 2022, American Chemical Society. d) Reproduced with permission.^[^ ^58^ ^]^ Copyright 2022, American Association for the Advancement of Science. e) Reproduced with permission.^[^ ^14^ ^]^ Copyright 2021, American Association for the Advancement of Science. f) Reproduced with permission.^[^ ^13^ ^]^ Copyright 2019, American Chemical Society. g) Reproduced with permission.^[^ ^29^ ^]^ Copyright 2022, Springer Nature.

At present, the structural ordering is being actively studied in mechanical materials to overcome the long‐standing trade‐off between strength and ductility to achieve superior mechanical properties, by constructing grain size and twin structure gradients and other ordered structures including layered structures.^[^ ^11^, ^22^, ^23^, ^24^, ^25^, ^26^ ^]^ In the field of functional materials, however, such study is at the infant stage and remains less explored.^[^ ^4^, ^19^, ^21^, ^27^, ^28^ ^]^ In this perspective paper, we discuss several representative cases in ordered functional materials, encompassing fabrication/assembly, structure, and properties as well as the mechanisms underlying their superior performance across catalytic, thermoelectric, and magnetic materials, see **Figure** **1**.^[^ ^13^, ^14^, ^17^, ^29^, ^30^, ^31^, ^32^ ^]^ Since the field of ordered functional materials is only at the infant state, a highly systematic discussion would be impossible. Nevertheless, we attempt to link the different applications by the common structural ordering characteristics across atomic‐, nano‐, and micro‐/macro‐scale, as well as the general physical mechanisms. Then, we show the idea of utilizing structural ordering to enhance materials properties or to create new functionalities has also emerged in some important research fields, such as neuromorphic computing and battery materials.^[^ ^33^, ^34^ ^]^ All these applications aim to generate and utilize energy more efficiently, which is one of the major challenges our society faces today and requires transformative materials. We hope these examples demonstrate the huge potential of ordered materials in solving such a challenge. We conclude by summarizing the general design principles, highlighting remaining scientific challenges, and making the prospects for the emerging ordered functional materials.

## 2 Ordered Catalytic Materials

Catalytic materials are essential to energy conversion technologies.^[^ ^27^, ^32^ ^]^ Catalytic reactions are governed by their thermodynamic energy barriers and kinetic processes and often suffer from a trade‐off between transport kinetics of reactant and local reaction kinetics.^[^ ^12^, ^13^, ^32^, ^35^ ^]^ Such a dilemma can be tackled by engineering ordered structures/assemblies that enable faster transport of reactants and/or larger reactant concentration around the reactive sites.^[^ ^12^, ^13^, ^36^ ^]^ Moreover, catalytic performance can also be enhanced through reducing the thermodynamic energy barrier of catalytic reactions by constructing ordered structures.

**Figure 2:**
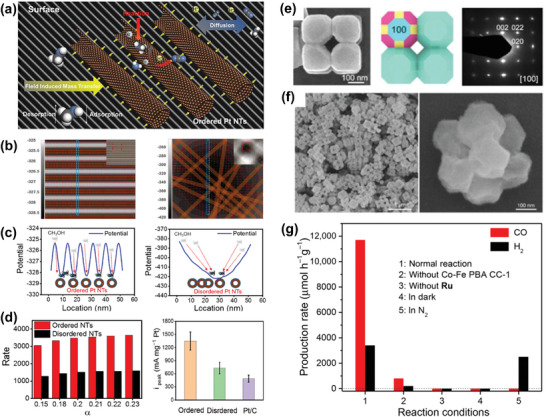
Structural ordering in catalytic materials. a) Schematic picture showing a periodic arrangement of Pt nanotubes on a glassy carbon electrode surface. b) Kinetic model simulations of the potential of methanol molecules around ordered (left) and disordered (right) Pt nanotubes. c) The cross section of the methanol potential showing periodic structure for ordered Pt nanotubes (left). For the disordered Pt nanotubes (right), the periodic potential structure collapses. d) Simulation (left) and experimental (right) comparisons of catalytic efficiency for ordered and disordered Pt nanotubes. a–d) Reproduced with permission.^[^ ^13^ ^]^ Copyright 2019, American Chemical Society. e) Field‐emission scanning electron microscopy (FESEM), schematic, and selected‐area electron diffraction (SAED) images of the Prussian blue analog nanocrystals with an ordered cubic structure. f) The cubic structure is stable after air annealing at 350 °C for 6 h. g) Comparison of the H_2_ and CO production rates of diverse catalysts with the ordered cubic structure (samples 2 and 3) and without the ordered cubic structure (samples 1, 4, and 5). e–g) Reproduced with permission.^[^ ^36^ ^]^ Copyright 2019, American Association for the Advancement of Science.

As shown in **Figure** **2**a, the nanoscale ordered catalytic Pt nanotubes were deposited in a periodic manner on the surface of a glassy carbon electrode by a self‐assembly method,^[^ ^13^ ^]^ and these Pt nanotubes can serve as active catalytic sites for methanol oxidation reaction. The comparison of methanol potential with ordered and disordered Pt nanotubes on the electrode surface is shown in Figure 2b, where the methanol molecules have lower potential close to the Pt nanotubes due to the existence of the microelectric field.^[^ ^13^ ^]^ Figure 2c shows the detailed cross‐section view of the potential profile of methanol molecules on the surface with ordered and disordered Pt nanotubes. When the Pt nanotubes are arranged in a periodic manner, the potential of methanol molecules has local peaks and valleys, and the local potential valleys locate at the Pt catalytic sites. This set of periodic potential will ensure the methanol molecules to be concentrated around the nearest Pt catalytic sites at the potential valleys. In this way, the methanol molecules will be distributed adequately around each Pt catalytic site which leads to maximized catalytic reaction efficiency. On the other hand, when the Pt nanotubes are formed in a random and disordered manner, the delicate periodic arrangement of the potential valleys collapses and only one global potential valley exists, which results in oversupply of methanol molecules at the global potential valley and insufficient of methanol molecules around Pt nanotubes elsewhere. As a result, the catalytic reaction efficiency is much higher for periodically arranged Pt nanotubes than that of disordered ones as shown both theoretically (Figure 2d left) and experimentally (Figure 2d right).

Nanoscale ordered cluster structures are also found effective in decreasing the thermodynamic energy barrier of catalytic reactions.^[^ ^36^ ^]^ Prussian blue analog nanocrystals can be constructed into single crystalline close‐packed cubic colloidal clusters with a chemical precipitation method,^[^ ^13^ ^]^ as shown by the field‐emission scanning electron microscopy (FESEM) and selected‐area electron diffraction (SAED) results in Figure 2e. Such a close‐packed cubic structure is stable and can retain its ordered structure after 350 °C annealing in air for 6 h, see Figure 2f. The catalytic performance of the ordered cluster structure is evaluated in the CO_2_ photoreduction experiment as shown in Figure 2g. Compared to the reaction without the ordered cluster structure, the ordered catalyst substantially increases the H_2_ and CO evolution reaction rate, which suggests the ordered cluster structures make considerable catalytic contribution to the H_2_ and CO conversion efficiency. Further analysis shows that the ordered cluster structures have good alignment of conduction band and valence band potentials, which increases the electron transition efficiency and facilitates the acceptance of photoexcited electrons in the CO_2_ reduction process.^[^ ^36^ ^]^

## 3 Ordered Thermoelectric Materials

Thermoelectric materials that can convert between thermal energy and electrical energy are critical for electric power generation and highly efficient cooling devices.^[^ ^5^ ^]^ Thermoelectric performance depends on the thermoelectric figure of merit $ZT\;=\frac{S^{2}\;\sigma }{k_{L}+k_{e}}\;$, where higher Seebeck coefficient (*S*) and electrical conductivity (*σ*), as well as lower lattice and electronic thermal conductivities (*k*_L_ and *k*_e_) are desired. However, these parameters often show conflicting relationships.^[^ ^5^ ^]^ It is therefore crucial to optimize one or more of them but not at the expense of others, which poses a great challenge in practical terms. More recently, structural ordering is identified as an effective method to optimize the conflicting properties simultaneously.^[^ ^14^, ^15^, ^37^, ^38^, ^39^, ^40^ ^]^

**Figure 3:**
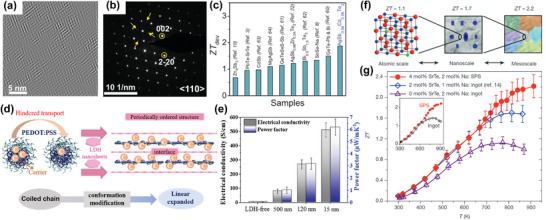
Structural ordering in thermoelectric materials. a) Local cation ordering can be observed in inverse fast Fourier transform of a high resolution scanning transmission electron microscopy (HRSTEM) high‐angle annular dark field (HAADF) image, and the nanoscale superstructure size (≈2–4 nm) is on the order of the mean free path of phonons (≈3–6 nm) in AgSbTe_2_. b) Selected area electron diffraction patterns, where the yellow arrows show signature of cationic order. c) Device figure of merit (*ZT*_dev_) of polycrystalline AgSb_0.94_Cd_0.06_Te_2_ with ordered structure. a–c) Reproduced with permission.^[^ ^14^ ^]^ Copyright 2021, American Association for the Advancement of Science. d) Schematic figure of the periodically ordered organic–inorganic hybrids. e) The reduced sizes of layered double hydroxides (LDHs) are proved to increase the interfacial surface‐to‐volume ratio and boost interfacial effect to intensify thermoelectric performance. d,e) Reproduced with permission.^[^ ^37^ ^]^ Copyright 2020, American Chemical Society. f) High *ZT* values with multiple scales of structural ordering. g) *ZT* values as a function of temperature for PbTe doped with 2 mol% Na (atomic scale), PbTe–SrTe (2 mol%) doped with 1 mol% Na (atomic scale plus nanoscale), and spark‐plasma‐sintered PbTe–SrTe (4 mol%) doped with 2% Na (atomic scale plus nanoscale and mesoscale). f,g) Reproduced with permission.^[^ ^15^ ^]^ Copyright 2012, Springer Nature.

For instance, at the atomic scale and nanoscale, the structural ordering can be utilized to achieve higher *ZT*.^[^ ^14^ ^]^ In pristine AgSbTe_2_, the cationic charge and size difference between Ag^+^ and Sb^3+^ are so large that it is not favorable for an ordered structure. However, dopant atoms Cd will preferentially occupy the disordered Sb sites rather than the ordered Sb sites to eliminate the Sb atoms in the disordered sites, and thus promotes the cationic ordering in AgSbTe_2_.^[^ ^14^ ^]^ The slight lattice constant differences between the nanoscale ordered superstructures and the main lattice will lead to local strain and cause strain ripples, where the phonon propagation can be greatly hindered, see **Figure** **3**a,b. Moreover, the doping of Cd can make the Fermi level far away from the mobility edge *E*_c_ (a critical energy), thus the electrical conductivity is improved by delocalizing electronic states. As a result, the thermoelectric performance can be significantly improved with a ZT value of 2.6 at 573 K, and the ordered thermoelectrics is one of the most advanced bulk thermoelectrics from room temperature to 573 K, see Figure 3c.

In addition, nanometer ordered layer structures, e.g., periodic organic–inorganic hybrids, can also be utilized to improve carrier mobility. Such ordered structures were constructed by alternately stacking inorganic 2D layered double hydroxides (LDHs) nanosheets and organic poly (3,4‐ethylenedioxythiophene)‐poly (styrene sulfonate) molecules through self‐assembly,^[^ ^37^ ^]^ expanding the interlayer channels to establish tunnels for carrier transport, see Figure 3d. Besides, the low‐energy carriers can be blocked at the boundary between the inorganic and the organic phase, while the high‐energy carriers can pass through, which means the low‐energy carriers will be filtered. Then the Seebeck coefficient and power factor of the ordered hybrids can be improved, and the thermoelectric performance is improved, see Figure 3e.

Moreover, the ordered structure can be constructed across multiple length scales to improve the *ZT* in the case of PbTe–SrTe. Multiscale hierarchically ordered architecture can be used to reduce the thermal conductivity through the modulation of atomic scale, nanoscale, and mesoscale phonon scattering in the spark‐plasma‐sintered (SPS) samples of PbTe–SrTe (4 mol%) doped with 2 mol% Na.^[^ ^15^ ^]^ The phonons scattered by the nanostructures of materials are mostly medium and short mean free path phonons, but when the additional mechanisms of grain‐boundary phonon scattering and impedance are introduced, the heat‐carrying phonons with longer mean free path can be effectively scattered. By integrating the effects of atomic‐scale alloy doping, endotaxial nanostructuring, and mesoscale grain boundaries, the maximum phonon scattering can be realized at high temperature, see Figure 3f. At the same time, the dissolution of sodium dopant can be enhanced at high temperature, and solid solution will reform with PbTe matrix, which can lower the Fermi level and increase the p‐type carrier density to improve the electrical conductivity, thus further improving the thermoelectric performance with a *ZT* value of 2.2 at 915 K, see Figure 3g. As such, through coordination of the ordered structures, we can improve the electrical conductivity and reduce the thermal conductivity simultaneously to enhance the thermoelectric performance. Constructing multiscale ordered structures will be a promising way to develop high‐performance thermoelectric materials.

## 4 Ordered Permanent‐Magnet Materials

Permanent‐magnet materials are essential to modern technologies, ranging from electric vehicles and wind power generators to ultrathin laptops.^[^ ^1^, ^8^ ^]^ Enhancing their magnetic properties are therefore of great significance to realize carbon neutrality for a future green society. The maximum energy product, (*BH*)_max_, is the key figure of merit to evaluate a permanent magnet that requires both high saturation magnetization (*M*_s_) and high coercivity (*H*_c_).^[^ ^7^ ^]^ However, there is an inherent trade‐off between the *M*_s_ and *H*_c_.^[^ ^19^, ^41^, ^42^ ^]^ Recently, structural orderings at atomic scale, nano scale, and microscale are found powerful in achieving a superior combination of high *M*_s_ and high *H*_c_, thus yielding strong magnets with higher (*BH*)_max_.^[^ ^16^, ^17^, ^43^, ^44^, ^45^ ^]^

**Figure 4:**
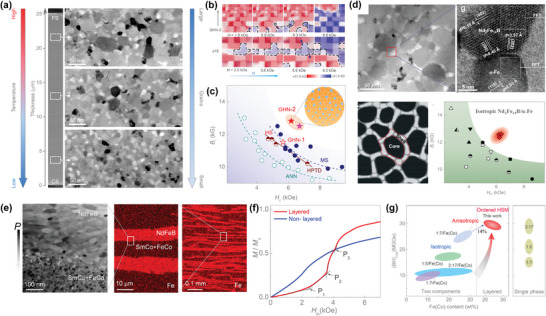
Structural ordering in magnetic materials. a) A grain size gradient resulted from a temperature gradient in the melt spinning process. b) Micromagnetic simulations of the magnetization reversal process with (top) and without (bottom) the grain size gradient. Without the grain size gradient, the magnetization reversal happens via a random and nonuniform manner, while a directional magnetization reversal is observed with the grain size gradient. c) A good combination of high remanent magnetization and high coercivity for the permanent magnet with the grain size gradient. a–c) Reproduced with permission.^[^ ^17^ ^]^ Copyright 2021, John Wiley and Sons. d) Nano scale core–shell structure and its effect in ensuring both high remanent magnetization and high coercivity. Reproduced with permission.^[^ ^47^ ^]^ Copyright 2016, American Chemical Society. e) Transmission electron microscopy (TEM) image and energy dispersive X‐ray (EDX) mapping of the micrometer scale (FeCo+SmCo)/NdFeB layered ordered structure. f) The multistep pinning magnetization mechanism observed in the (FeCo+SmCo)/NdFeB layered ordered structure. g) High energy product of the layered ordered (FeCo+SmCo)/NdFeB nanocomposite magnet. e–g) Reproduced with permission.^[^ ^45^ ^]^ Copyright 2018, John Wiley and Sons.

A nanoscale gradient was discovered effective in decoupling the trade‐off between the *M*_s_ and *H*_c_ of nanocomposite magnets. In conventional nanocomposite magnets comprising hard and soft‐magnetic components, e.g., Nd_2_Fe_14_B/a‐Fe, with a disordered and homogeneous structure, the magnetic reversal happens in a random manner and starts from the “weak points” with smaller nucleation field for reverse domains,^[^ ^41^ ^]^ e.g., defects and the soft a‐Fe phase. This nonuniform magnetization reversal is usually accompanied by cooperative cluster reversal of many nanograins, deteriorating both the *H*_c_ and hysteresis loop squareness of the magnets, see **Figure** **4**b. Recently, by engineering an ordered structure with grain‐size gradient in the Nd_2_Fe_14_B/a‐Fe nanocomposites using a temperature‐gradient‐assisted self‐assembly strategy (Figure 4a),^[^ ^17^ ^]^ a unique directional magnetization reversal occurs to counter the cooperative cluster‐grain reversal observed in the conventional disordered nanocomposites, see Figure 4b. The directional magnetization reversal starts from larger grains toward smaller grains, as the exchange coupling between the hard‐ and soft‐magnetic phases is stronger for smaller grains and, at the same time, smaller grains have a higher nucleation field for reverse domains,^[^ ^41^ ^]^ see Figure 4b. This new directional magnetization reversal can effectively suppress the cooperative cluster‐grain reversal and enables a rare combination of large *M*_s_, high *H*_c_, and large loop squareness, thus yielding a record‐high energy product (26 MGOe) for isotropic permanent‐magnet materials, see Figure 4c.

More recently, by engineering a nanoscale ordered *α*‐Fe/Pr_2_Fe_14_B nanocomposite with reverse gradients in both the grain size and *α*‐Fe content via the temperature‐gradient‐field self‐assembly,^[^ ^16^ ^]^ a record energy product of 25 MGOe for isotropic *α*‐Fe/Pr_2_Fe_14_B magnets has been achieved. Further optimizing such reverse gradients enables a nearly uniform magnetization reversal critical to ultrahigh energy product without sacrificing *M*_s_, which is unattainable with existing methods.^[^ ^16^ ^]^

The core–shell nanostructure is another example of nanoscale ordering to enhance the exchange coupling between magnetic functional components.^[^ ^46^, ^47^ ^]^ As shown in Figure 4d, a core–shell ordered nanostructure with Nd_2_Fe_14_B cores and a‐Fe shells were fabricated by self‐assembly in alloy melt.^[^ ^47^ ^]^ In such ordered structure, sufficient exchange coupling between the hard‐magnetic Nd_2_Fe_14_B phase and the soft‐magnetic a‐Fe phase enables a good combination of high remanent magnetization (*B*_r_) and high *H*_c_, see Figure 4d. As a result, a high energy product (25 MGOe) was yielded, with 30% less consumption of expensive rare‐earth metals than pure rare‐earth magnets.

Extending the structural ordering beyond nanoscale, an ordered layer structure at the micrometer scale also shows great potential to enhance the energy product of permanent magnets.^[^ ^45^ ^]^ Such layered ordered structures were constructed by a reductionist strategy, that is, creating macroscopic materials through the controlled assembly of small building blocks. When (FeCo+SmCo) and NdFeB layers are arranged in a periodic structure (Figure 4e), the exchange coupling between the hard and soft magnetic phases is enhanced and, more importantly, a new multistep pinning mechanism emerges, where multiple pinning fields are observed in magnetization processes, see Figure 4f. This new multistep pinning mechanism effectively counters the undesired cooperative cluster‐grain reversal and enables an increase in *H*_c_ without sacrificing both the *B*_r_ and hysteresis loop squareness. As a result, the highest energy product of 31 MGOe is obtained up to now for the low‐rare‐earth magnets (containing more than 20 wt% of cheap soft‐magnetic phase, e.g., a‐Fe).^[^ ^45^ ^]^ This value is comparable to that of commercial SmCo magnets, but the ordered nanostructure consumes 20–30 % less expensive rare‐earth metals, see Figure 4g.

## 5 Emerging Ordered Functional Materials in Neuromorphic Computation and Battery

Recently, the noteworthy roles of structural ordering in enhancing materials properties or creating new functionalities have started to raise attention in some important fields, such as neuromorphic computation^[^ ^33^, ^48^ ^]^ and battery materials.^[^ ^34^, ^49^, ^50^, ^51^, ^52^ ^]^

**Figure 5:**
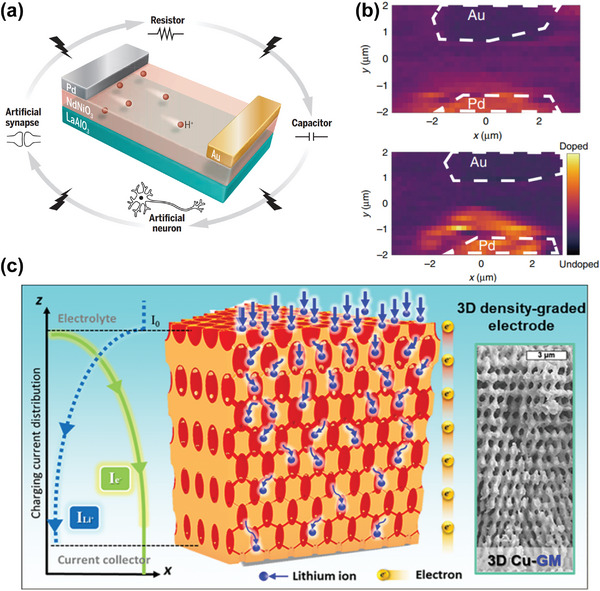
Emerging materials with ordered structures. a) By modulating the proton gradient in the channel of NdNiO_3_ device, multiple artificial neural functions can be achieved such as artificial synapses and artificial neurons. Reproduced with permission.^[^ ^58^ ^]^ Copyright 2022, American Association for the Advancement of Science. b) Visualization of the proton gradient near the Pd electrode through the local X‐ray absorption spectroscopy (XAS) technique. Reproduced with permission.^[^ ^48^ ^]^ Copyright 2020, Springer Nature. c) Density‐graded composite electrodes with a porosity gradient and expected total current (*I*_0_) distribution within the electrode. Reproduced with permission.^[^ ^59^ ^]^ Copyright 2022, American Chemical Society.

The neuromorphic computation aims to perform efficient computational tasks with less energy using the principles of human neural network. Simulating neural functions with artificial neurons and artificial synapses is therefore required. Recently, it is reported that hydrogen doping can trigger a Mott electronic metal to insulator phase transformation in rare‐earth perovskite nickelates, which can be utilized to simulate neural functions.^[^ ^48^, ^53^, ^54^, ^55^, ^56^ ^]^ More importantly, the hydrogen dopants create an ordered concentration gradient at atomic scale in the device channel, and modulating the gradient via electric pulses leads to different occupations of the proton metastable states as well as different artificial brain‐like functions, which is critical to highly efficient dynamic neural network computations, see **Figure** **5**a,b.^[^ ^33^, ^57^, ^58^ ^]^

For sodium ion battery anode materials, high sodium ion diffusion coefficient is important to ensure good battery performances. Recently, it is reported that the sodium ion diffusion can be facilitated in an ordered hierarchical self‐assembly of metal‐phenolic mesocrystals.^[^ ^51^ ^]^ In addition, electrodes with ordered structures are also proposed to be beneficial to prevent local mechanical damage and prolong battery life.^[^ ^34^ ^]^ For example, density‐graded composite electrodes with a porosity gradient at micrometer scale were discovered to be effective in reducing energy loss at high charging rates by mitigating polarization,^[^ ^59^ ^]^ as shown in Figure 5c. Moreover, electrodes made of particles with a size gradient can suppress the nonuniform kinetics and corresponding local mechanical damage to maintain high chemical activity of the battery.^[^ ^34^, ^49^, ^50^, ^52^ ^]^

## 6 Challenges and Prospects

Despite the diversity of functional materials, engineering their ordered structures shares a common design principle: structural ordering enables collective behaviors that could create disruptive properties or new functionalities far beyond the reach of existing materials. Such collective response can stem from the unprecedented coupling effect among the ordered units at a single scale or across multiple length scales, yielding significant impacts on phonon energy dispersion, electronic band structure, and the thermodynamics and kinetics of functional processes.^[^ ^3^ ^]^ Based on this viewpoint, we believe, like natural materials, structural ordering strategy should be general and applicable to most functional materials, as unambiguously demonstrated in recent studies across catalytic, thermoelectric, magnetic, neuromorphic computing, and battery materials.^[^ ^13^, ^14^, ^16^, ^33^, ^34^, ^35^, ^59^ ^]^ Indeed, besides these examples demonstrated above, the structural ordering was also found effective in improving other functionalities such as optical property, ionic conductivity, and multiferroic characteristics.^[^ ^29^, ^31^, ^60^ ^]^

However, ordered functional materials are still in the infancy. Many scientific and technological challenges remain to be resolved to fully unleash their potential to create revolutionary properties. First, the engineered ordered structures are often limited to the systems comprising only one or two types of structural units and most ordered structures were built at the single length scale, such as atomic, nano, or microscale. Creating ordered functional materials with multiple components across various length scales, like natural materials, will enable us to fully exploit the benefits provided by the ordered structures to achieve disruptive properties or functionalities. To meet this goal, new processing techniques need to be devised such as additive manufacturing and field‐assisted assembly/synthesis methods. Moreover, both the reductionist strategy and the rational combination of bottom‐up and top‐down approaches as well as multiple fabrication technologies are also promising to make such complex multiscale ordered structures. Second, new technologies are required to fabricate 3D macroscopic ordered structures, while maintaining a rigorous control over the structural characteristics of constituent units including size, concentration/content, and orientation. Third, scientific advances in understanding the relationships among processing, structure, and properties are essential for developing the emerging ordered functional materials, not only for their structural design but also for practical applications. Meeting these demands needs transformative processing strategies; moreover, cooperative iterations between experiments and simulations are required. In this aspect, theoretical simulations are particular useful in understanding the ordered structure–property relationships^[^ ^3^ ^]^ as well as the complex coupling effects at multiple length scales among the ordered units.^[^ ^3^ ^]^ However, multiscale modeling remains a tough challenge for existing methods. Last but not least, large scale production of ordered functional materials at low costs is also necessary for future industrial applications. All these are quite challenging tasks. As such, close multi‐disciplinary collaborations among physicists, chemists, and materials scientists are imperatively required to develop the emerging ordered functional materials.

In spite of these challenges, engineering ordered structures that were selected by natural materials through billions of years of evolution opens up unprecedented opportunities to push the performance limits of existing functional materials. First, building ordered functional materials enables us to overcome the inherent conflicts among their essential physical properties that limit materials performance. This achievement is obtained by introducing new physical mechanisms stemming from structural ordering in functional processes, such as the reactant redistribution optimization in ordered catalytic materials, the low‐energy carrier blocking in ordered thermoelectrics, and the directional magnetization reversal in ordered permanent‐magnet materials. As such, amplified properties and new functionalities could be realized by building ordered structures. Furthermore, different from conventional compositional design strategy, constructing ordered materials takes a structural design strategy that does not rely on alloying or addition of expensive critical elements, thus making the materials cheaper and more sustainable. Even more importantly, this unique architecture strategy enables us to break though the constraint of relying on chemical elements (e.g., alloying) to enhance materials performances, thus opening up new opportunities for materials development. Finally, the structural ordering provides a new dimension for material design. With this new dimension, materials properties can be efficiently modulated by additional degrees of freedom and, more importantly, many long‐standing conflicting properties could be decoupled, thus leading to disruptive materials with superior properties unattainable in existing materials. We hope this perspective can timely attract the attention of the scientific community to the emerging ordered functional materials and trigger intense studies on this topic.

## Conflict of Interest

The authors declare no conflict of interest.
